# Appearance anxiety and college students’ exercise participation: a chain mediation model of negative body image and exercise self-efficacy

**DOI:** 10.3389/fpsyg.2026.1828308

**Published:** 2026-06-01

**Authors:** Yifan Zhang, Jiaqi Hao, Kai Guo, Qian Huang, Lvhao Liu

**Affiliations:** Wuhan Sports University, Wuhan, China

**Keywords:** appearance anxiety, chain mediation model, exercise participation, exercise self-efficacy, negative body image

## Abstract

**Objective:**

This study explores the relationship between appearance anxiety (AA) and exercise participation and constructs a chain mediation model through the mediating effects of negative body image (NBI) and exercise self-efficacy.

**Methods:**

This study employed the Appearance Anxiety Scale-Brief Version, the Negative Physical Self Scale-General Appearance Subscale (NPSS-G), the Self-efficacy for Exercise (SEE) scale, and the Physical Activity Rating Scale (PARS-3) to conduct a questionnaire survey among Chinese college students. A total of 1,145 questionnaires were distributed, and 1,052 valid responses were collected, yielding an effective response rate of 91.88%. Structural equation modeling (SEM) and the bootstrap method were used to analyze the relationships among the variables and the mediating effects, with statistical significance set at *p* < 0.05.

**Results:**

The results revealed that appearance anxiety was significantly negatively correlated with exercise participation and exercise self-efficacy and significantly positively correlated with negative body image. Both negative body image and exercise self-efficacy significantly mediated the relationship between appearance anxiety and participation in exercise, and together, they formed a significant chain-mediating relationship.

**Discussion:**

The findings of this study indicate a chain-linked association pattern among appearance anxiety, negative body image, exercise self-efficacy, and exercise participation that aligns with the theoretical model, suggesting that appearance-related negative cognitions may be connected to exercise participation through body image evaluation and exercise belief. In practice, while promoting exercise participation, universities should concurrently emphasize diverse aesthetic education, body acceptance support, the enhancement of exercise self-efficacy, and the optimization of the exercise context to strengthen the sustainability of college students’ exercise participation.

## 1 Introduction

In recent years, within an environment characterized by highly visual social media and persistent social comparison, college students have increasingly come to regard physical appearance as a crucial basis for social evaluation, thereby heightening their level of concern and anxiety regarding their image (Ruan et al., 2025). In 2021, a targeted survey of Chinese university students revealed that 59.03% of respondents experienced a certain degree of appearance anxiety (AA) concerning their facial features or body shape (Xue and Yu, 2022), indicating that appearance anxiety has emerged as a significant issue in the domain of student mental health. Concurrent with this, exercise participation, a vital behavioral avenue for promoting physical and mental well-being as well as social interaction among college students, remains suboptimal in terms of actual engagement (Sun et al., 2025). In this context, there is an urgent need to investigate the relationship between appearance anxiety and college students’ exercise participation, along with the underlying psychological pathways, to provide a foundation for targeted body acceptance education, exercise support interventions, and mental health promotion initiatives within higher education institutions.

Existing research has revealed the adverse consequences of appearance anxiety from multiple perspectives, encompassing not only psychological aspects such as self-esteem (Liao et al., 2023), social anxiety, and depression (Patel et al., 2025), and loneliness (Chang et al., 2026), but also behavioral aspects such as eating disorders (Jin et al., 2022) and social avoidance (Guo and Wu, 2023). Furthermore, existing research indicates that appearance anxiety is not only associated with body image flexibility (Acar, 2022) and body dissatisfaction (Ahmad and Safdar, 2025) but may also influence self-efficacy (Liao et al., 2023), while body image (Sabiston et al., 2019; Gao et al., 2025) and exercise self-efficacy (Zhao et al., 2023; Sheng et al., 2025) are considered important psychological factors influencing exercise participation. Thus, the relationship between appearance anxiety and exercise participation among college students is likely not a simple direct association but rather operates through interconnected psychological mechanisms such as body image and self-efficacy. However, the majority of the existing studies remain limited to examining pairwise relationships or single pathways between variables, and there is a lack of a systematic explanation of how appearance anxiety jointly influences college students’ exercise participation through negative body image and exercise self-efficacy. Accordingly, to overcome the limitations of previous studies that only examined indirect effects in isolation, this study adopted the SOR analytical framework to construct a chain mediation model. This framework better integrates the relationships among situational stimuli, individual psychological states, and behavioral outcomes, and incorporates appearance anxiety, negative body image, exercise self-efficacy, and exercise participation into a unified analytical framework.

The heightened level of appearance anxiety among college students may be significantly associated with their excessive attention to and comparison of others’ lives and appearances on social media. Appearance anxiety (AA) primarily refers to an individual’s worry regarding how others evaluate their physical appearance and represents an appearance-evaluation-oriented anxiety experience (Ayhan et al., 2022). Research indicates that appearance anxiety is a common emotional disturbance among college students, and it is significantly negatively associated with exercise participation (Liu et al., 2024). Specifically, individuals with appearance anxiety may feel concerned or embarrassed about exposing their bodies in gyms or other group exercise settings (Cowley and Schneider, 2025), leading them to choose non-group activities or avoid exercise participation altogether to circumvent exposing their bodies in front of others (Taylor and Gill, 2004). On this basis, this study proposes the following hypothesis:

*H1*: Appearance anxiety is significantly negatively correlated with exercise participation among college students.

As a mediating effect examined in this study, negative body image (NBI) refers to an individual’s negative cognitive and affective evaluation of his or her own body shape and overall physical self, emphasizing an internal negative representation of the body (Song and Zhu, 2019). Although negative body image and appearance anxiety are closely related, they are not equivalent. The former foregrounds “negative cognition regarding the body”, whereas the latter emphasizes “anxiety about being evaluated”. Research has indicated a significant negative correlation between appearance anxiety and exercise participation, and a negative body image may be a critical psychological variable for elucidating this relationship (Mahdifar et al., 2024; Jiang and Wang, 2025). First, individuals have an inherent tendency to evaluate themselves through comparisons with others (Festinger, 1954), which leads them to frequently contrast themselves with idealized images portrayed in the media and further contributes to negative body image (Festino et al., 2024). Second, excessive preoccupation with body image can link to avoidance behaviors (Zakaria et al., 2022), which are associated with lower levels of exercise participation (Sabiston et al., 2019; Marquez et al., 2024). On the basis of the preceding analysis, this study proposes the following hypothesis:

*H2*: Negative body image mediates the relationship between appearance anxiety and exercise participation among college students.

Exercise self-efficacy (ESE) refers to an individual’s belief and determination to overcome difficulties and persist in exercise participation when confronted with physical challenges (McAuley and Blissmer, 2000). As a key variable in understanding the relationship between appearance anxiety and college students’ exercise participation, exercise self-efficacy plays an important explanatory role in this link. According to Bandura’s self-efficacy theory, appearance anxiety, as a negative emotional experience, not only affects an individual’s psychological state but is also associated with lower levels of exercise self-efficacy (Bandura, 1977). Furthermore, there is a close intrinsic association between an individual’s exercise self-efficacy and exercise participation (McAuley et al., 2011; Choi et al., 2017). Higher exercise self-efficacy is significantly positively correlated with an individual’s level of exercise participation, whereas lower exercise self-efficacy is significantly negatively correlated with an individual’s level of exercise participation (Ouyang et al., 2020). On this basis, the following hypothesis is proposed:

*H3*: Exercise self-efficacy mediates the relationship between appearance anxiety and exercise participation among college students.

Existing research suggests that higher levels of appearance anxiety are often accompanied by elevated levels of negative body image and diminished exercise self-efficacy, and that both negative body image and exercise self-efficacy are, in turn, significantly associated with exercise participation (Ouyang et al., 2020; Liao et al., 2023; Zhao et al., 2023). Individuals with higher levels of appearance anxiety tend to be more attentive to the gaze of others and exhibit heightened sensitivity to being watched or evaluated, a tendency that may become more pronounced in gyms, swimming pools, or other group exercise settings (Focht and Hausenblas, 2004; Park and Pinkus, 2009). When individuals frequently compare their appearance with idealized images, they are more likely to magnify the discrepancy between their actual body and the ideal standard, thereby leading to more negative body evaluations (Bizman et al., 2001). In exercise settings characterized by a degree of public exposure, display, and evaluability, the negative bodily feelings by college students can undermine their confidence in exercise participation and be linked to lower levels of exercise self-efficacy (Zhou et al., 2025), which is associated with lower exercise participation. On this basis, this study proposes the following hypothesis:

*H4*: Negative body image and exercise self-efficacy play a chain mediating role in the relationship between appearance anxiety and exercise participation among college students.

## 2 Methods

### 2.1 Participants and procedure

**Figure 1:**
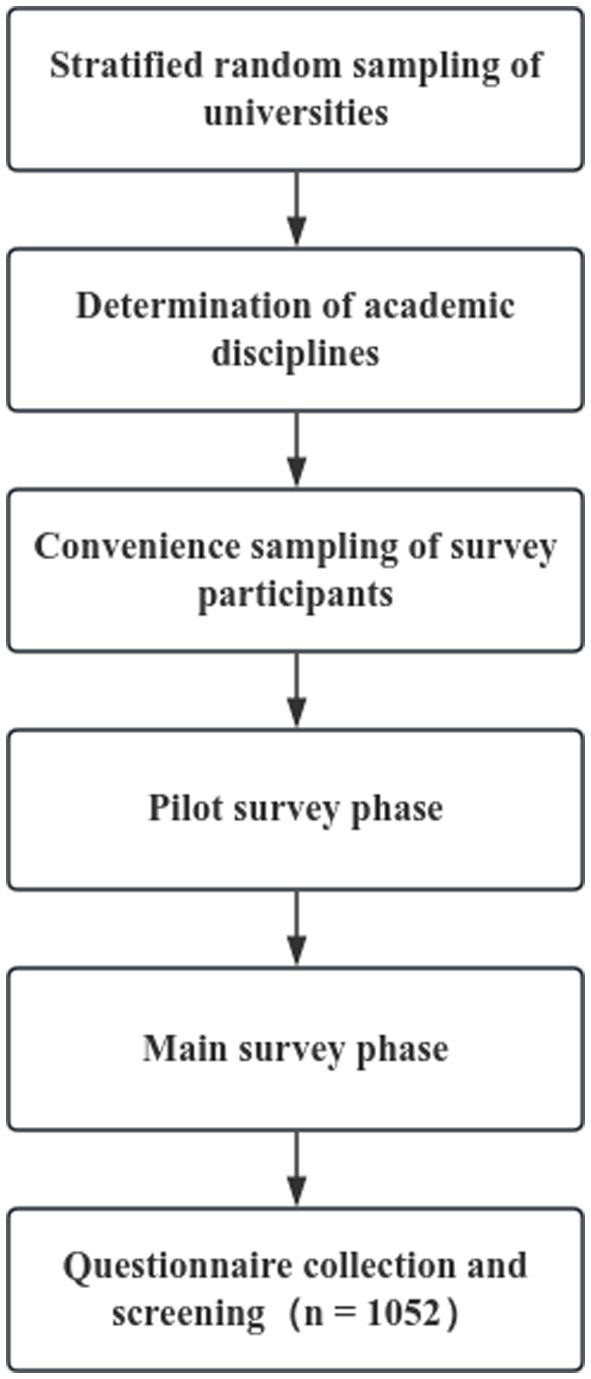
Sample selection process.

This study employed a combination of stratified random sampling and convenience sampling. Specifically, first, universities nationwide were divided into two categories: “985/211” universities and “non-985/211” universities. Six universities were randomly selected from each category, with each selected university comprising students from the following disciplines: science and engineering; literature, history, philosophy, and law; economics and management; medicine; arts and physical education; and other. Second, convenience sampling was employed to distribute questionnaires to undergraduate students across these disciplinary categories, utilizing a combination of online platforms (such as WeChat and QQ) and offline methods (such as paper-based distribution and collection). The entire survey process was divided into two main phases: during the pilot survey phase, 80 eligible respondents were selected to test the questionnaire’s reliability and validity, as well as the clarity of the item wording; during the formal survey phase, a total of 1,145 questionnaires were distributed, and 1,052 valid responses were ultimately collected, resulting in a response rate of 91.88%. Figure 1 illustrates the sample selection process.

In this survey, responses with missing data, anomalous data, or evident patterned answers were excluded on the basis of the following criteria: (1) If a substantial proportion of core variable items were left unanswered (e.g., exceeding 10% of the core items in the respective questionnaire), the response was deemed invalid. (2) If the same option was selected consecutively for 15 or more items within the core scales and lacked reasonable content variation, the response was classified as patterned answering. (3) This study did not include standalone attention-check questions, nor did it exclude questionnaires based solely on a single response-duration threshold. Instead, exclusion decisions were made through a comprehensive assessment that combined patterns of missing data, extended runs of identical responses, and the logical consistency of the answers provided. Overall, the sample in this study comprises college students of different genders, academic years, disciplines, and types of institutions, indicating a certain degree of sample heterogeneity that provides a data foundation for subsequent analyses. The detailed sample information is presented in Table 1.

**Table 1:** Descriptive statistics of the sample.

Item	Categories	Frequency	Percentage (%)
Gender	Male	550	52.3
	Female	502	47.7
Grade	Freshman	195	18.5
	Sophomore	219	20.8
	Junior	158	15
	Senior	255	24.2
	Postgraduate and above	225	21.4
Place of origin	Urban area	586	55.7
	Rural area	466	44.3
Major	Science and Engineering	296	28.1
	Literature, History, Philosophy, Law	235	22.3
	Economics and Management	241	22.9
	Medicine	191	18.2
	Art and Physical Education	68	6.5
	Other	21	2
University type	“985”/“211” Universities	401	38.1
	Non-985/211 universities	651	61.9
Relationship status	Yes (In a relationship)	401	38.1
	No (Not in a relationship)	651	61.9
Monthly disposable income	Below 1,000 Yuan	183	17.4
	1,001 ~ 1,500 Yuan	256	24.3
	1,501 ~ 2,000 Yuan	415	39.4
	2,001 ~ 3,000 Yuan	134	12.7
	Above 3,000 Yuan	64	6.1
Height (cm)	157 ~ 161	205	19.5
	162 ~ 166	199	18.9
	167 ~ 171	220	20.9
	172 ~ 176	198	18.8
	177 ~ 182	230	21.9
Weight (kg)	46 ~ 51	192	18.3
	52 ~ 57	216	20.5
	58 ~ 63	212	20.2
	64 ~ 69	203	19.3
	70 ~ 76	229	21.8

Table 1 presents the descriptive statistics of the demographic information of the survey respondents, covering nine aspects: gender, grade, major, university type, place of origin, relationship status, monthly disposable income, height, and weight. First, the gender distribution of the sample was relatively balanced, with males accounting for 52.3% and females accounting for 47.7%, indicating a slightly lower proportion of female participants than male participants. The distribution across the five academic-year cohorts was relatively even, with the largest group being fourth-year students, totaling 255 individuals, and representing 24.2% of the sample. With respect to the place of origin, the proportions of students from urban and rural areas were comparable, accounting for 55.7 and 44.3%, respectively.

Second, in terms of students’ academic enrollment, the distribution across majors was uneven. Students majoring in science and engineering constituted the largest proportion (28.1%). With respect to institution type, the collected sample encompassed two tiers: “985/211” universities and “non-985/211” universities. The majority of the respondents were from “non-985/211” universities, with 401 students (38.1%) from “985/211” universities and 651 students (61.9%) from “non-985/211” universities. With respect to monthly disposable income, the sample data were predominantly concentrated in the range of 1,001–2,000 Renminbi (RMB), with the highest proportion (39.4%) falling within the 1,501–2,000 RMB bracket. In terms of relationship status, the majority of respondents were single, with the proportion of single individuals (61.9%) significantly exceeding that of those in a relationship. Finally, the distribution across the five height intervals of the sample population was relatively balanced, with a small coefficient of variation. The 177–182 cm interval contained the greatest number of individuals, accounting for 21.9% of the total. The distribution across weight intervals was also relatively even, with the 70–76 kg interval having the highest number of respondents (229 individuals), comprising 21.8% of the sample. Overall, the distribution pattern across various demographic dimensions was fairly balanced, demonstrating a certain degree of sample heterogeneity and coverage, which provided a data foundation for subsequent analyses.

### 2.2 Conceptual framework

This study draws upon the stimulus-organism-response (SOR) analytical framework to construct a model to elucidate the relationships among variables associated with college students’ exercise participation in the context of appearance-evaluation pressure. Compared with traditional mediation models that merely examine the statistical transmission relationships among variables, the SOR framework places greater emphasis on the structural connections among situational cues, internal psychological processing, and behavioral responses (Mehrabian and Russell, 1974; Jacoby, 2002); consequently, it is more suitable for organizing the overarching relational structure among appearance anxiety, negative body image, exercise self-efficacy, and exercise participation examined in this study. In recent years, the SOR framework has been applied to explain the relationships among external stimuli, individual internal states, and behavioral responses in the domain of exercise-related behavior (Song et al., 2022). From the perspective of the present study, the “stimulus” can be understood as the background of appearance-evaluation pressure faced by college students, encompassing contextual conditions such as social comparison, idealized appearance standards, the sense of being watched, and potential evaluative cues within exercise settings. Appearance anxiety represents the proximal emotional response formed by an individual under such background conditions and is entered into the analysis as the initial variable in the empirical model. Negative body image and exercise self-efficacy reflect an individual’s internal psychological state in terms of his or her cognitive evaluation of the body and beliefs about exercise capability and are therefore situated at the “organism” level. The level of college students’ exercise participation manifests as a behavioral outcome associated with these psychological processes.

**Figure 2:**
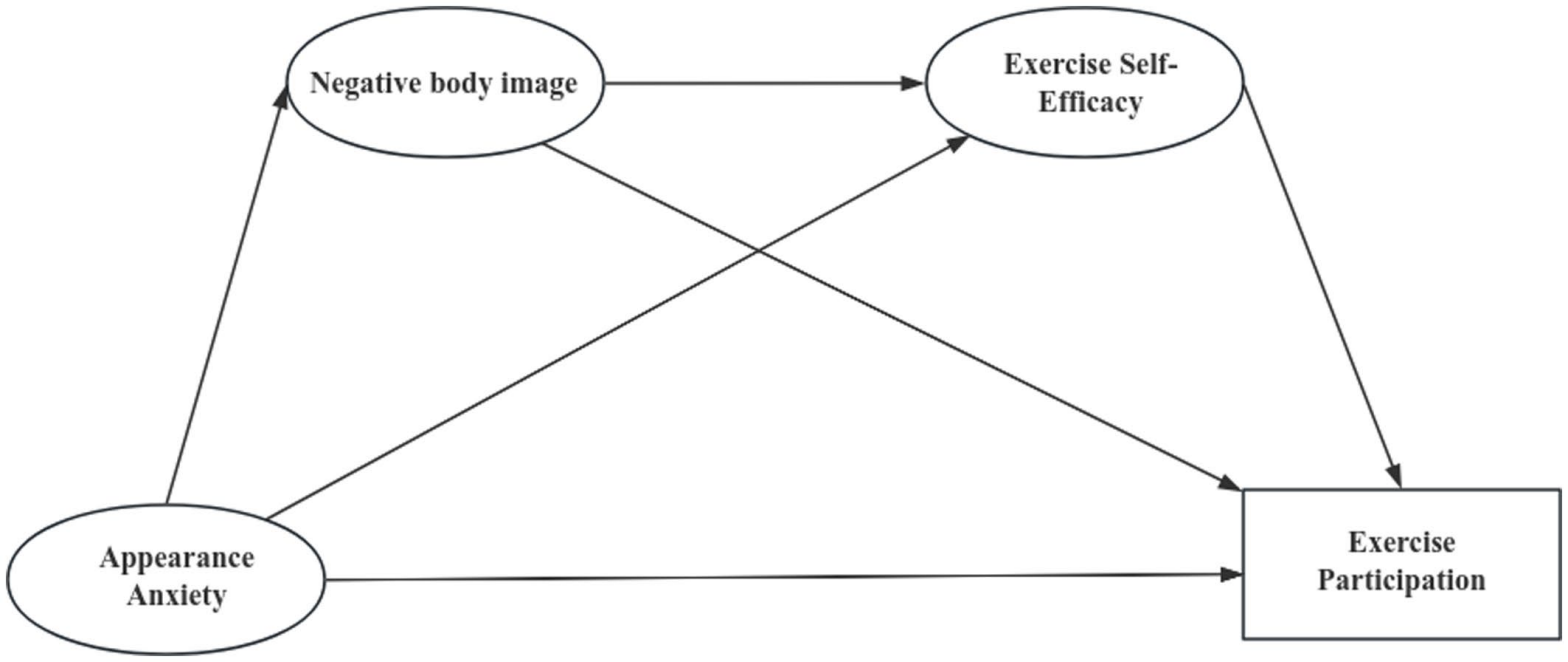
Framework of the model.

On the basis of the aforementioned rationale, this study designates appearance anxiety as the independent variable, exercise participation as the dependent variable, and negative body image and exercise self-efficacy as the mediating variables, thereby constructing a chain mediation model. Given that cross-sectional data were employed in this study, the specified variable sequence should be interpreted as a prioritized explanation consistent with the theoretical model rather than as an empirical confirmation of a unidirectional causal relationship. The specific framework of the chain mediation model is shown in Figure 2.

### 2.3 Measures

This study employed established and validated scales from relevant fields to measure four variables: appearance anxiety, negative body image, exercise self-efficacy, and exercise participation.

Appearance anxiety was measured using the Appearance Anxiety Scale-Brief Version, developed by Dion et al. (1990), which comprises 14 items rated on a 5-point scale ranging from 1 (strongly disagree) to 5 (strongly agree). Positively worded items were reverse-coded during data processing, so that higher scores indicated a higher level of appearance anxiety in the individual.

Negative body image was primarily assessed using the Negative Physical Self Scale-General Appearance Subscale (NPSS-G) developed by Chen et al. (2006), which measured adolescents’ dissatisfaction with their physical selves. In accordance with existing research, the NPSS may be administered using specific subscales in isolation depending on the research objectives. For example, Yan et al. (2025) utilized the Negative Physical Self Scale-Facial Appearance Subscale (NPSS-A) in a sample of college students. He et al. (2025) used the Negative Physical Self Scale-General Appearance Subscale (NPSS-G) to measure negative body image in their study. Given that the original wording of these items reflected positive body evaluation, they were reverse coded during data processing so that higher scores indicate a higher level of negative body image. In this study, the Cronbach’s alpha of this scale was 0.868.

Exercise self-efficacy was measured using the Self-efficacy for Exercise (SEE) scale (Resnick and Jenkins, 2000). This scale assesses an individual’s confidence in persisting with exercise in various situations that pose barriers to Exercise participation. It consists of nine items, with higher scores indicating a greater degree of exercise confidence.

Exercise participation was measured by drawing on the work of Zhang and Li (2021) and utilizing the Physical Activity Rating Scale (PARS-3) to assess college students’ levels of exercise participation. This scale evaluates an individual’s exercise participation on the basis of the intensity, duration, and frequency of physical exercise and calculates a total score according to the classic scoring formula: Exercise Participation Level = Exercise Intensity × (Exercise Duration - 1) × Exercise Frequency. Higher scores indicate higher levels of exercise participation. In subsequent analyses, the total score computed on the basis of the aforementioned formula was used as the observed variable for exercise participation in the structural model. Given that the PARS-3 primarily measures the frequency, duration, and intensity of individual exercise behavior, “exercise participation” in this study primarily refers to college students’ level of engagement in exercise rather than broader forms of sports spectatorship, sports consumption, or other indirect forms of participation.

### 2.4 Statistical analysis

In this study, Statistical Package for the Social Sciences (SPSS) 30.0, Analysis of Moment Structures (AMOS) 29.0, and R (lavaan package) were used for data processing and statistical analyses. Specifically, SPSS 30.0 was used primarily for sample organization, preliminary data screening, and descriptive statistical analysis of demographic characteristics. AMOS 29.0 was used mainly for confirmatory factor analysis (CFA), measurement model fit testing, reliability and validity analyses, competitive model comparison, and structural path estimation. R (lavaan package) was used for bias-corrected bootstrap testing of mediating effects and for outputting total, direct, and individual indirect effect parameters. Mediation analysis was conducted using 5,000 bootstrap resamples, confidence intervals set at 95%, and statistical significance determined at *p* < 0.05.

First, a descriptive statistical analysis of the sample’s demographic characteristics was performed. Second, the normality of the data was examined prior to structural equation modeling (SEM). Univariate normality was assessed using the skewness and kurtosis for each observed variable, whereas multivariate normality was evaluated using the multivariate kurtosis coefficient and its critical ratio (CR) from the AMOS output. The results indicated that the skewness and kurtosis values of all the observed variables fell within acceptable ranges, suggesting that the data largely met the assumption of normality. The multivariate normality test revealed a degree of deviation. Considering the relatively large sample size of this study and the Likert-scale nature of the questionnaire data, which are distributional characteristics commonly encountered in related research, this deviation was deemed unlikely to substantially influence subsequent analyses.

Building on this foundation, a confirmatory factor analysis (CFA) was conducted to test the measurement model. The reliability and validity of the scales were evaluated using standardized factor loadings, composite reliability (CR), average variance extracted (AVE), and Cronbach’s *α* coefficients.

To further examine the discriminant validity between appearance anxiety and negative body image, a competitive confirmatory factor analysis was performed in addition to baseline measurement model testing. Specifically, a three-factor model was constructed by specifying appearance anxiety, negative body image, and exercise self-efficacy as three distinct latent variables. An alternative two-factor model was also constructed in which all measurement items for appearance anxiety and negative body image were combined into a single latent variable, whereas exercise self-efficacy was retained as a separate latent variable. Model comparison relied primarily on indices such as the χ^2^/df, the comparative fit index (CFI), the Tucker–Lewis index (TLI), the root mean square error of approximation (RMSEA), and information criteria (Akaike Information Criterion [AIC] and Bayesian Information Criterion [BIC]). If the three-factor model demonstrated a substantially superior fit compared with the two-factor model, it would indicate that treating appearance anxiety and negative body image as two distinguishable constructs better aligns with the data. On the basis of a satisfactory measurement model fit, a structural equation model was subsequently constructed to estimate the pathways associated with appearance anxiety, negative body image, exercise self-efficacy, and exercise participation, which is consistent with the theoretical model.

In the structural model, appearance anxiety, negative body image, and exercise self-efficacy were entered as latent variables, and exercise participation was represented by the total score calculated using the classic PARS-3 formula, which was entered as an observed variable. For brevity, the total exercise participation score derived from the classic PARS-3 formula is denoted as EP in subsequent tables and model path diagrams.

## 3 Results

### 3.1 Measurement model

To verify the reliability and validity of the research data, this study employed confirmatory factor analysis to assess the measurement model’s fit and adequacy. The core indices selected for this evaluation included the χ^2^/df, goodness-of-fit index (GFI), adjusted goodness-of-fit index (AGFI), normed fit index (NFI), Tucker–Lewis index (TLI), comparative fit index (CFI), and root mean square error of approximation (RMSEA). These indices reflect the quality of the model fit from different dimensions. The results indicated that the measurement model exhibited a good overall fit. Specifically, χ^2^/df = 1.963, GFI = 0.954, AGFI = 0.946, NFI = 0.960, TLI = 0.978, CFI = 0.980, and RMSEA = 0.030, all of which met commonly accepted thresholds, indicating that the measurement model possessed a satisfactory fit. The detailed information is presented in Table 2.

**Table 2:** Measurement model fit indices.

Indicators	χ^2^/df	GFI	AGFI	NFI	TLI	CFI	RMSEA
Results	1.963	0.954	0.946	0.960	0.978	0.980	0.030
Standards	<3	>0.9	>0.9	>0.9	>0.9	>0.9	<0.05

After the measurement model fit reached acceptable standards, we examined the reliability and convergent validity of each latent variable. As shown in Table 3, within the main sample (*N* = 1,052), the standardized factor loadings for all the items of appearance anxiety, negative body image, and exercise self-efficacy exceeded 0.70. The composite reliability (CR) values for the three constructs were 0.941, 0.889, and 0.912, respectively, all exceeding the threshold of 0.70. The average variance extracted (AVE) values were 0.587, 0.586, and 0.564, respectively, all surpassing the criterion of 0.50. The Cronbach’s *α* coefficients were 0.949, 0.868, and 0.922, respectively, all of which met acceptable benchmarks. These results indicate that the internal consistency and convergent validity of the measurement model are satisfactory. Exercise participation, calculated as a total score based on the classic PARS-3 formula, was entered as an observed variable in the subsequent structural model analysis; therefore, its factor loadings, CR, AVE, and Cronbach’s α are not reported.

**Table 3:** Questionnaire items.

Variable	Item	Load	SD	CR	AVE	Cronbach’s α
Appearance anxiety	AA1: I feel nervous about certain aspects of my appearance.	0.781	1.164	0.941	0.587	0.949
	AA2: I worry about how others evaluate my appearance.	0.748				
	AA3: I am satisfied with my appearance.	0.748				
	AA4: I like the way I look.	0.729				
	AA5: I want to change my appearance.	0.702				
	AA6: I am satisfied with my body shape.	0.748				
	AA7: I feel uncomfortable with certain aspects of my appearance.	0.784				
	AA8: I feel that most of my friends are more attractive than me in terms of appearance.	0.723				
	AA9: I hope I can look good.	0.740				
	AA10: I worry about my ability to attract a romantic partner.	0.766				
	AA11: I feel comfortable with my appearance.	0.779				
	AA12: I am satisfied with my weight.	0.759				
	AA13: When others comment on my appearance, I feel nervous.	0.761				
	AA14: I believe others will find my appearance attractive.	0.764				
Negative body image	NBI1: I feel proud of my body.	0.781	1.159	0.889	0.586	0.868
	NBI2: I like my body very much.	0.748				
	NBI3: Overall, I am satisfied with my body.	0.753				
	NBI4: I have no regrets about my body.	0.765				
	NBI5: There is nothing about my body that needs to be changed.	0.776				
Exercise self-efficacy	ESE1: Even if the weather bothers me, I still feel confident to exercise.	0.739	1.170	0.912	0.564	0.922
	ESE2: Even if I am not interested in the exercise, I still feel confident to do it.	0.718				
	ESE3: Even if I feel pain during exercise, I still feel confident to continue.	0.768				
	ESE4: Even if I exercise alone without a companion, I still feel confident to do it.	0.752				
	ESE5: Even if I do not feel the joy of exercise, I still feel confident to continue.	0.755				
	ESE6: Even if I am busy with other things, I still feel confident to exercise.	0.756				
	ESE7: Even if I feel tired, I still feel confident to exercise.	0.774				
	ESE8: Even if I feel stressed, I still feel confident to exercise.	0.715				
	ESE9: Even if I feel depressed, I still feel confident to exercise.	0.798				

Items for negative body image were reverse-coded; higher scores indicate higher levels of global negative body evaluation. Exercise participation was computed using the PARS-3 formula and modeled as an observed variable; therefore, factor loadings, CR, AVE, and Cronbach’s α were not reported for this variable.

In terms of discriminant validity, as presented in Table 4, the square roots of the AVE for each latent variable were greater than their respective correlation coefficients with other variables. Specifically, the square roots of the AVE for appearance anxiety, negative body image, and exercise self-efficacy were 0.766, 0.765, and 0.751, respectively, all of which exceeded the absolute values of the correlation coefficients among these constructs. This finding indicates that the latent variables possess satisfactory discriminant validity and that the measurement model demonstrates good differentiation.

**Table 4:** Discriminant validity among latent constructs.

Variable	AA	NBI	ESE
AA	0.766		
NBI	0.459	0.765	
ESE	−0.412	−0.419	0.751

The diagonal values represent the square roots of the AVE, and the off-diagonal values represent the correlation coefficients between latent variables.

To examine whether appearance anxiety and negative body image are empirically distinguishable, an additional comparison between the hypothesized three-factor measurement model and an alternative two-factor model was conducted. As shown in Table 5, the three-factor model exhibited a good fit (χ^2^/df = 2.015, CFI = 0.980, TLI = 0.978, RMSEA = 0.031, AIC = 817.187, BIC = 1109.735), whereas the two-factor model demonstrated a markedly poorer fit (χ^2^/df = 7.250, CFI = 0.876, TLI = 0.866, RMSEA = 0.077, AIC = 2644.227, BIC = 2926.859). Compared with the two-factor model that combined appearance anxiety and negative body image into a single construct, the three-factor model provided a substantially better fit to the sample data. This suggests that despite the moderate correlation between appearance anxiety and negative body image, treating them as two distinct latent variables aligns more closely with the data structure, further supporting the discriminant validity between the two constructs.

**Table 5:** Comparison of alternative measurement models.

Model	χ^2^/df	CFI	TLI	RMSEA	AIC	BIC
Three-factor model (AA, NBI, and ESE)	2.015	0.980	0.978	0.031	817.187	1,109.735
Two-factor model (AA+NBI and ESE)	7.250	0.876	0.866	0.077	2,644.227	2,926.859

In the two-factor model, appearance anxiety and negative body image were combined into a single latent factor, whereas exercise self-efficacy remained a separate latent factor.

### 3.2 Structural model

In this study, covariance-based structural equation modeling (SEM) was employed to examine the pathways associated with the variables, which is consistent with the theoretical model (Jöreskog, 1978); model fit was assessed, and path relationships were verified using AMOS 29.0 software. A higher degree of model fit indicates better congruence between the theoretical hypotheses and actual sample data.

All indicators in Table 2 met the commonly accepted fit criteria in academic research, indicating that the theoretical model adequately fit the sample data. On this basis, the path coefficients were analyzed to determine the estimated results and significance of the pathways associated with appearance anxiety, negative body image, exercise self-efficacy, and exercise participation (Table 6).

**Table 6:** Path test results.

Path	B	SE	C. R.	*p*	β
AA–EP	−4.835	1.031	−4.688	***	−0.161
AA–NBI	0.436	0.034	12.975	***	0.459
AA–ESE	−0.253	0.032	−7.818	***	−0.278
NBI–EP	−4.599	1.144	−4.020	***	−0.146
NBI–ESE	−0.279	0.036	−7.832	***	−0.292
ESE–EP	8.620	1.141	7.556	***	0.261

***p < 0.001.

**Figure 3:**
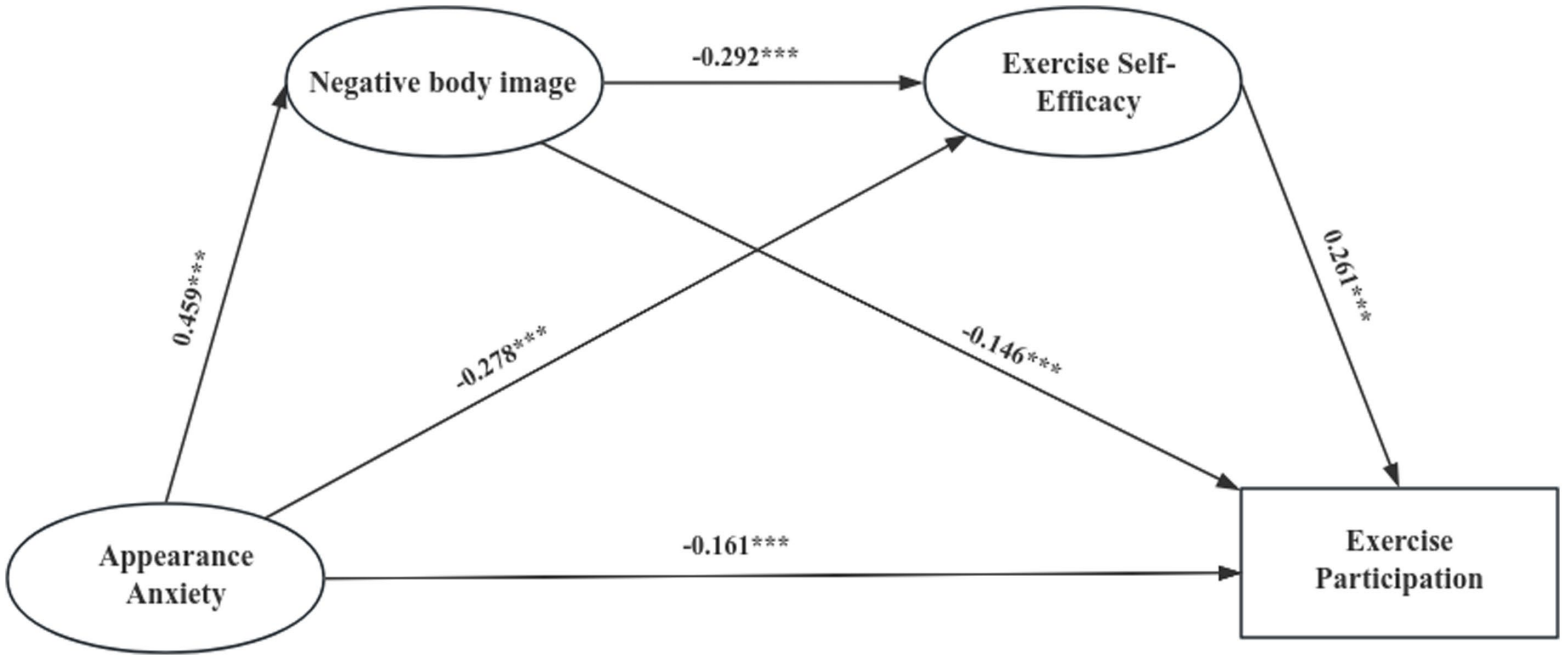
Path coefficients of the conceptual model. ***p < 0.001.

Table 6 presents the structural path estimates of the hypothesized model, including unstandardized coefficients (B), standard errors (SE), critical ratios (CR), *p*-values, and standardized coefficients (*β*). The results of the data analysis indicated that appearance anxiety was significantly positively correlated with negative body image (β = 0.459, *p* < 0.001). In addition, appearance anxiety was significantly negatively correlated with exercise self-efficacy (β = −0.278, *p* < 0.001) and exercise participation (β = −0.161, *p* < 0.001). Notably, the significant negative association between appearance anxiety and exercise participation supports H1. In terms of the magnitude of the standardized path coefficients, the strength of the association between appearance anxiety and negative body image was greater than that between appearance anxiety and exercise participation. This may be attributable to the fact that negative body image and appearance anxiety are conceptually more proximate to appearance-related cognitive-emotional experiences, thus resulting in a stronger relationship. In contrast, exercise participation is influenced by various factors, including time availability, physical ability, environmental conditions, and social support, which may explain its relatively weak association with appearance anxiety. Furthermore, negative body image was significantly negatively correlated with exercise self-efficacy (β = −0.292, *p* < 0.001) and exercise participation (β = −0.146, *p* < 0.001). Finally, exercise self-efficacy was significantly and positively correlated with exercise participation (β = 0.261, *p* < 0.001). The path coefficients in the structural model described above are shown in Figure 3.

### 3.3 Mediation analysis

To further examine the mediating role of negative body image and exercise self-efficacy in the relationship between appearance anxiety and college students’ participation in exercise, this study employed the bias-corrected bootstrap method to test the mediating effects of appearance anxiety and college students’ participation in exercise. The analysis was conducted using 5,000 bootstrap resamples and 95% confidence intervals. Specific mediating effect parameters were generated using the lavaan package in R.

As shown in Table 7, the total effect of appearance anxiety on college students’ participation in exercise was significant (effect = −10.215, 95% bias-corrected confidence interval (BC CI) = [−12.270, −8.273], *p* < 0.001). After controlling for the mediating variables, the direct effect of appearance anxiety on college students’ participation in exercise remained significant (effect = −4.905, 95% BC CI [−7.172, −2.685], *p* < 0.001), indicating a significant association between appearance anxiety and college students’ participation in exercise. Moreover, the total indirect effect was significant (effect = −5.310, 95% BC CI [−6.763, −3.978], *p* < 0.001), accounting for 52.0% of the total effect, suggesting that negative body image and exercise self-efficacy played significant mediating roles in the relationship between appearance anxiety and college students’ participation in exercise. Specifically, the indirect effect via negative body image was significant (AA–NBI–EP: effect = −2.035, 95% BC CI [−3.262, −0.980], *p* < 0.001), accounting for 19.9% of the total effect, thereby supporting H2. The indirect effect via exercise self-efficacy was significant (AA–ESE–EP: effect = −2.211, 95% BC CI [−3.286, −1.422], *p* < 0.001), accounting for 21.6% of the total effect, thereby supporting H3. The chain mediating effect via negative body image and exercise self-efficacy was also significant (AA–NBI–ESE–EP: effect = −1.064, 95% BC CI [−1.622, −0.702], *p* < 0.001), accounting for 10.4% of the total effect, thereby supporting H4.

**Table 7:** Results of the mediating effect test.

Path	Effect value	SE	*p*	Bias-corrected 95% CI		Ratio %
				Lower	Upper	
Total effect	−10.215	1.029	***	−12.270	−8.273	100
Direct effect	−4.905	1.131	***	−7.172	−2.685	48.0
Indirect effects	−5.310	0.716	***	−6.763	−3.978	52.0
AA–NBI–EP	−2.035	0.583	***	−3.262	−0.980	19.9
AA–ESE–EP	−2.211	0.466	***	−3.286	−1.422	21.6
AA–NBI–ESE–EP	−1.064	0.228	***	−1.622	−0.702	10.4

***p < 0.001. Bootstrap-based unstandardized estimates of the total, direct, and indirect effects.

## 4 Discussion and recommendations

### 4.1 Discussion

This study examined the relationship between appearance anxiety and exercise participation among a sample of college students and further tested the independent mediating effects of negative body image and exercise self-efficacy, as well as their chain mediating effect. Given the cross-sectional self-report data utilized in this study, the following discussion should be understood solely as an “associational interpretation” consistent with the theoretical model rather than as a strict inference of causal mechanisms or unidirectional transmission processes. Furthermore, the ensuing discussion concerning “negative body image” should be interpreted as referring specifically to the general dimension of negative body image captured by the abbreviated measurement described above.

1. This study revealed a significant negative association between appearance anxiety and exercise participation among college students. These findings are largely consistent with the results of Li et al. (2025) and Liu et al. (2025a, b). Appearance anxiety and social physique anxiety do not necessarily motivate individuals to adopt more proactive body management behaviors (Liu et al., 2024); rather, they may weaken their willingness to engage in exercise and even induce exercise avoidance by heightening concerns about social evaluation and situational tension (Li et al., 2025). College students with elevated appearance anxiety tend to be more concerned about how they appear in the eyes of others, fear being watched or evaluated, and worry about exposing perceived bodily flaws in public exercise settings, thereby exhibiting stronger intentions of social withdrawal (Park and Pinkus, 2009). Moreover, negative emotions such as anxiety and shyness stemming from concerns about one’s appearance may not only affect an individual’s level of participation but also influence their choice of exercise activities (Sabiston et al., 2019; Aktağ et al., 2021; Festino et al., 2024).
2. This study further revealed that negative body image mediates the relationship between appearance anxiety and participation in exercise. Specifically, the stronger an individual’s appearance anxiety is, the more likely they are to develop negative bodily perceptions and dissatisfaction evaluations, and such a negative body image, in turn, undermines their level of exercise participation. These findings are largely consistent with the work of Sabiston et al. (2019). College students tend to be overly concerned with their appearance, which may intensify their focus on and magnification of perceived physical flaws, thereby increasing the risk of depression and negative emotions (Liu et al. (2025a, b)). They may further transform localized dissatisfaction into a systemic negative body image (Turel et al., 2018). Negative body image heightens individuals’ concerns about how others judge their appearance and performance, thereby reinforcing feelings of shame, tension, and avoidance (Cowley and Schneider, 2025). Higher levels of body dissatisfaction are generally associated with a lower frequency and poorer sustainability of exercise participation (Marquez et al., 2024). Furthermore, body dissatisfaction among college students may stem from a broader system comprising psychological, behavioral, and lifestyle factors. Existing research indicates that body image-related experiences often co-occur with dietary quality and sleep behaviors (Papale et al., 2025). At the same time, physical activity is not only related to energy expenditure but may also promote more positive health and psychological outcomes by enhancing body image satisfaction (Condello et al., 2019). Further mediation studies also suggest that there may be continuous associative pathways among physical activity, body weight, and body image satisfaction (Condello et al., 2016). Therefore, future research could adopt a more comprehensive, multidimensional approach to assess body dissatisfaction and elucidate its mechanisms of action in greater detail.
3. In this study, exercise self-efficacy mediated the relationship between appearance anxiety and exercise participation. Individuals with higher levels of appearance anxiety may experience greater negative emotions, which can lead them to subjectively magnify the difficulty of exercise tasks and indirectly influence their level of exercise self-efficacy (Brunet and Sabiston, 2009). Self-efficacy is associated not only with the initiation of exercise but also with adherence to exercise over time (McAuley, 1993). When individuals doubt their ability to successfully complete exercise tasks or present themselves appropriately in front of others, their exercise motivation and behavioral persistence tend to be lower (Rhodes et al., 1999). Thus, among college students, this psychological pressure from appearance anxiety can transform exercise from a pursuit of health or personal interest into a social context characterized by comparison and evaluation, thereby undermining their exercise self-efficacy and further reducing their level of exercise participation.
4. An important finding of this study is that negative body image was significantly negatively correlated with exercise self-efficacy. This is consistent with the findings of Soulliard et al. (2019). Body image-related issues are associated with an individual’s willingness to participate in physical activity (Ramos-Jiménez et al., 2017; Sabiston et al., 2019), while a positive body image is associated with increased confidence in exercise and more positive subjective exercise experiences (Soulliard et al., 2019). Focusing on the mechanism of appearance anxiety, negative body image, exercise self-efficacy, and exercise participation, appearance anxiety increases negative body image among college students, and this increase in negative body image is accompanied by a decrease in exercise self-efficacy. These results suggest that the relationships among appearance anxiety, negative body image, and exercise participation may not exist in isolation but rather exhibit a chain mediating relationship. When individuals experience elevated levels of appearance anxiety, they are more likely to compare their actual bodies with idealized physical standards, and this sense of discrepancy is generally associated with a more negative body image (Turel et al., 2018), which further undermines their exercise self-efficacy, leading to a decline in their motivation and persistence in physical activity.

### 4.2 Policy recommendations

On the basis of the aforementioned conclusions, this study proposes the following recommendations to promote exercise participation among college students:

1. The integration of “diverse aesthetics and body acceptance” within campus mental health education should be strengthened, and negative body image should be considered as a cognitive dimension. Given the significant associations observed in this study between appearance anxiety, negative body image, and exercise participation, universities should reduce the reinforcement of singular aesthetic standards and increase the dissemination of content emphasizing the functional value of the body through mental health education and campus culture initiatives. The cognitive pressure associated with being evaluated or compared may be alleviated through thematic courses, group counseling sessions, and awareness campaigns.
2. Enhance exercise self-efficacy within physical education instruction and training organizations by combining “incremental task progression with immediate feedback”, thereby facilitating the accumulation of mastery experiences. Given that exercise self-efficacy is significantly positively correlated with exercise participation and plays a notable role in the chain mediation relationship, physical education curricula should adopt more extensively stratified goal-setting and process-oriented assessments. By establishing attainable short-term objectives, providing timely positive reinforcement, and documenting progress, students can be better supported in cultivating a stable sense of control and competence in their learning.
3. Optimize the context of exercise participation by reducing public exposure and social evaluation cues, thereby enhancing the sense of psychological safety when individuals enter exercise settings. Considering that individuals with higher levels of appearance anxiety and negative body image are more likely to perceive public exercise environments as threatening evaluative contexts, universities may mitigate students’ sense of being watched and the anticipation of embarrassment by establishing “nonpublic, self-service exercise spaces” and “evaluation-free” activities. Examples include constructing single-person fitness pods, providing 24-h self-service gyms, and offering more welcoming beginner-level classes and low-visibility activity options. Such measures can lower the entry threshold for populations with low exercise self-efficacy.
4. Mechanisms for peer support and cooperative physical activities to enhance the sense of belonging and sustained engagement during exercise should be established. Exercise participation is not solely an individual behavior but also has salient social and contextual attributes. By forming mutual support groups, peer-buddy systems, collaborative projects, and supportive communities, students’ sense of belonging and sustained investment in physical exercise may be strengthened. In practical terms, this provides more supportive conditions for students with a higher negative body image or lower exercise self-efficacy, thereby increasing both the likelihood and the sustainability of their exercise participation.

### 4.3 Limitations and future research

The limitations of this study are as follows, which also suggest directions for future research.

1. This study employed a cross-sectional survey design. Although this design allows for examining whether the relationships among appearance anxiety, negative body image, exercise self-efficacy, and exercise participation align with the theoretical model, it cannot establish strict causal ordering or capture dynamic change processes among these variables. In turn, participation in exercise may influence an individual’s negative body image and exercise self-efficacy. Moreover, alternative sequences or bidirectional reciprocal relationships may exist among negative body image, exercise self-efficacy, and exercise participation. Therefore, the directional specification of the variables in this study is primarily grounded in theoretical deduction and should not be interpreted as an empirical confirmation of a unidirectional relationship. Future research could employ longitudinal tracking or experimental designs to further examine the temporal sequence and stability of the associations among these variables.
2. All the data in this study were derived from self-report questionnaires. Although procedural controls were implemented through anonymous responses and reverse scoring, the findings may still be susceptible to social desirability effects and common method bias. Future research should incorporate peer evaluations, behavioral indicators, or objective exercise data to increase the robustness of the measurement outcomes.
3. This study primarily examined the chain-mediating roles of negative body image and exercise self-efficacy without incorporating other important variables that may influence exercise participation, such as social support, sex differences, exercise interest, and the exercise environment. Consequently, the model results presented in this study should primarily be understood as an associative pattern consistent with the theoretical framework rather than as net effect estimates derived under conditions of full control for other relevant variables. The potential for omitted variable bias remains and warrants further investigation in future studies through the inclusion of additional covariates, adoption of longitudinal designs, or more rigorous model specifications. Furthermore, the formal survey primarily relied on convenience sampling, resulting in certain limitations regarding the representativeness of the sample. Future research should conduct validation studies across a broader range of university types and student populations and incorporate more individual and contextual factors to increase the explanatory power and generalizability of the research conclusions.
4. This study did not utilize the full Negative Physical Self Scale (NPSS); instead, it operationalized the measurement of negative body image using the Negative Physical Self Scale-General Appearance Subscale (NPSS-G). Although this abbreviated measure demonstrated good internal consistency and structural validity in the present study, and additional reliability, correlational, it captures only a portion of the negative body image construct and should not be equated with a comprehensive measurement of the full, multidimensional construct.

## 5 Conclusion

Based on a sample of Chinese college students, this study examined the relationship between appearance anxiety and exercise participation. It tested the mediating roles of negative body image and exercise self-efficacy. The results indicate that appearance anxiety is significantly negatively correlated with exercise participation; simultaneously, negative body image and exercise self-efficacy play significant independent mediating roles in this relationship, further forming a significant chain mediation pathway. Overall, this study elucidates the association between appearance anxiety and exercise participation from the dual perspectives of body cognition and exercise beliefs, providing a reference for universities to implement body acceptance education, optimize exercise environments, and enhance students’ exercise self-efficacy.

## Data Availability

The raw data supporting the conclusions of this article will be made available by the authors, without undue reservation.

## References

[ref1] Acar S. (2022). Mediator role of social appearance anxiety in the relationship between socio-cultural attitudes towards appearance and body image flexibility. Int. J. Psychol. Educ. Stud. 9, 332–339. doi: 10.52380/ijpes.2022.9.2.481

[ref2] Ahmad G. Safdar Z. S. (2025). Body shaming and body dissatisfaction with mediating role of social appearance anxiety among women university students with below and above average body mass index. Pak. J. Psychol. Res. 40, 461–482. doi: 10.33824/PJPR.2025.40.2.27

[ref3] Aktağ I. Akbulut M. K. Tuzcuoğlu S. (2021). Social appearance anxiety and leisure time exercise level of high school students. Eur. J. Educ. Stud. 8, 352–363. doi: 10.46827/ejes.v8i4.3704

[ref4] Ayhan H. Savsar A. Yilmaz Sahin S. Iyigun E. (2022). Investigation of the relationship between social appearance anxiety and perceived social support in patients with burns. Burns 48, 816–823. doi: 10.1016/j.burns.2021.08.020, 34521565

[ref5] Bandura A. (1977). Self-efficacy: toward a unifying theory of behavioral change. Psychol. Rev. 84, 191–215. doi: 10.1037/0033-295X.84.2.191, 847061

[ref6] Bizman A. Yinon Y. Krotman S. (2001). Group-based emotional distress: an extension of self-discrepancy theory. Personal. Soc. Psychol. Bull. 27, 1291–1300. doi: 10.1177/01461672012710005

[ref7] Brunet J. Sabiston C. M. (2009). Social physique anxiety and physical activity: a self-determination theory perspective. Psychol. Sport Exerc. 10, 329–335. doi: 10.1016/j.psychsport.2008.11.002

[ref8] Chang J. Guo D. Dai S. (2026). The impact of appearance anxiety on loneliness among college students: an integrative model. BMC Psychol. 14, 548. doi: 10.1186/s40359-026-04269-z, 41808224 PMC13088726

[ref9] Chen H. Jackson T. Huang X. (2006). The negative physical self scale: initial development and validation in samples of chinese adolescents and young adults. Body Image 3, 401–412. doi: 10.1016/j.bodyim.2006.07.005, 18089244

[ref10] Choi J. Lee M. Lee J. Kang D. Choi J.-Y. (2017). Correlates associated with participation in physical activity among adults: a systematic review of reviews and update. BMC Public Health 17:356. doi: 10.1186/s12889-017-4255-2, 28438146 PMC5404309

[ref11] Condello G. Capranica L. Migliaccio S. Forte R. Baldassarre A. D. Pesce C. (2019). Energy balance and active lifestyle: potential mediators of health and quality of life perception in aging. Nutrients 11, 2122 doi: 10.3390/nu11092122, 31489886 PMC6770584

[ref12] Condello G. Capranica L. Stager J. Forte R. Falbo S. Baldassarre A. D. . (2016). Physical activity and health perception in aging: do body mass and satisfaction matter? A three-path mediated link. PLoS One 11:e0160805. doi: 10.1371/journal.pone.0160805, 27611689 PMC5017576

[ref13] Cowley E. S. Schneider J. (2025). “I sometimes feel like I can’t win!”: an exploratory mixed-methods study of women’s body image and experiences of exercising in gym settings. PLoS One 20:e0316756. doi: 10.1371/journal.pone.0316756, 39879151 PMC11778772

[ref14] Dion K. L. Dion K. K. Keelan J. P. (1990). Appearance anxiety as a dimension of social-evaluative anxiety: exploring the ugly duckling syndrome. Contemp. Soc. Psychol. 14, 220–224.

[ref15] Festinger L. (1954). A theory of social comparison processes. Hum. Relat. 7, 117–140. doi: 10.1177/001872675400700202

[ref16] Festino E. Papale O. Rocco F. D. Maio M. D. Cortis C. Fusco A. (2024). Effect of physical activity behaviors, team sports, and sitting time on body image and exercise dependence. Sports 12, 260. doi: 10.3390/sports12090260, 39330737 PMC11435772

[ref17] Focht B. C. Hausenblas H. A. (2004). Perceived evaluative threat and state anxiety during exercise in women with social physique anxiety. J. Appl. Sport Psychol. 16, 361–368. doi: 10.1080/10413200490517968

[ref18] Gao X. Wang X. Chee C. S. Bin Samsudin S. Hassan M. Z. Ma L. . (2025). Predictive model of the relationship between social support, body image perception, and physical activity among university students. Humanit. Soc. Sci. Commun. 12:809. doi: 10.1057/s41599-025-04854-4

[ref19] Guo Q. Wu M. (2023). The relationship between self-objectification and social avoidance among Chinese middle adolescent girls: the mediating role of appearance comparison and self-esteem. Curr. Psychol. 42, 3489–3497. doi: 10.1007/s12144-021-01705-8

[ref20] He D. Gilcharan Singh H. K. Alavi M. Koo H. C. Fariduddin M. N. Wee L. H. . (2025). Body image dissatisfaction and disordered eating behaviors in chinese female undergraduate students: the mediating role of emotional regulation strategies. J. Eat. Disord. 13:104. doi: 10.1186/s40337-025-01287-x, 40474276 PMC12139299

[ref21] Jacoby J. (2002). Stimulus-organism-response reconsidered: an evolutionary step in modeling (consumer) behavior. J. Consum. Psychol. 12, 51–57. doi: 10.1207/S15327663JCP1201_05

[ref22] Jiang Y. Wang X. (2025). The effects of physical activity on social physique anxiety in college students—the mediating and moderating role of mental toughness and negative physical self. BMC Psychol. 13:54. doi: 10.1186/s40359-025-02377-w, 39827341 PMC11748591

[ref23] Jin Y. Xu S. Chen C. Wilson A. Gao D. Ji Y. . (2022). Symptom association between social anxiety disorder, appearance anxiety, and eating disorders among chinese university students: a network analysis to conceptualize comorbidity. Front. Public Health 10:1044081. doi: 10.3389/fpubh.2022.1044081, 36620231 PMC9814491

[ref24] Jöreskog K. G. (1978). Structural analysis of covariance and correlation matrices. Psychometrika 43, 443–477. doi: 10.1007/BF02293808

[ref25] Li Q. Shao L. Li H. Zhu Y. Li Y. (2025). Associations between weight stigma and exercise avoidance motivation among college students: exploring the roles of internalized weight stigma and social anxiety. Front. Psychol. 16, 1655699. doi: 10.3389/fpsyg.2025.1655699, 41190128 PMC12581209

[ref26] Liao J. Xia T. Xu X. Pan L. (2023). The effect of appearance anxiety on social anxiety among college students: sequential mediating effects of self-efficacy and self-esteem. Behav. Sci. 13:692. doi: 10.3390/bs13080692, 37622832 PMC10451712

[ref27] Liu Z. Fu M. Shi J. Hu Y. Gao X. (2025b). The psychological mechanism of self-objectification: the interaction between sociocultural pressures and the self-system. Front. Psychol. 16, 1531222. doi: 10.3389/fpsyg.2025.1531222, 41089645 PMC12517067

[ref28] Liu L. Liu G. Wang H. (2024). Self-objectification and appearance anxiety in university students: physical activity as a moderator. Soc. Behav. Personal. Int. J. 52, 1–9. doi: 10.2224/sbp.13340

[ref29] Liu X. Soh K. G. Lu Y. (2025a). Effect of dance on social physique anxiety and physical self-esteem among adults: a systematic review. Front. Psychol. 16, 1547802. doi: 10.3389/fpsyg.2025.1547802, 40535192 PMC12174144

[ref30] Mahdifar M. Sany S. B. T. Tehrani H. Ghavami V. Shahroodi M. V. (2024). Body image perception and physical activity behavior among adult population: application of trans-theoretical model of behavior change. PLoS One 19:e0297778. doi: 10.1371/journal.pone.0297778, 38408055 PMC10896515

[ref31] Marquez B. Zhang X. Huang X. Mendoza-Vasconez A. Benitez T. Marcus B. (2024). Body image and physical activity in latinas. J. Behav. Med. 47, 531–536. doi: 10.1007/s10865-024-00472-8, 38393444 PMC11234904

[ref32] McAuley E. (1993). Self-efficacy and the maintenance of exercise participation in older adults. J. Behav. Med. 16, 103–113. doi: 10.1007/BF00844757, 8433355

[ref33] McAuley E. Blissmer B. (2000). Self-efficacy determinants and consequences of physical activity. Exerc. Sport Sci. Rev. 28, 85–88. 10902091

[ref34] McAuley E. Mullen S. P. Szabo A. N. White S. M. Wójcicki T. R. Mailey E. L. . (2011). Self-regulatory processes and exercise adherence in older adults: executive function and self-efficacy effects. Am. J. Prev. Med. 41, 284–290. doi: 10.1016/j.amepre.2011.04.014, 21855742 PMC3160622

[ref35] Mehrabian A. Russell J. A. (1974). An Approach to Environmental Psychology. Cambridge, MA, US: The MIT Press.

[ref36] Ouyang Y. Wang K. Zhang T. Peng L. Song G. Luo J. (2020). The influence of sports participation on body image, self-efficacy, and self-esteem in college students. Front. Psychol. 10:3039. doi: 10.3389/fpsyg.2019.03039, 32116869 PMC7012809

[ref37] Papale O. Festino E. Rocco F. D. Maio M. D. Foster C. Cortis C. . (2025). Eating right, sleeping tight? A cross-sectional study on the student-athlete paradox for diet and sleep behaviors. Nutrients 17, 2946. doi: 10.3390/nu17182946, 41010473 PMC12472328

[ref38] Park L. E. Pinkus R. T. (2009). Interpersonal effects of appearance-based rejection sensitivity. J. Res. Personal. 43, 602–612. doi: 10.1016/j.jrp.2009.02.003

[ref39] Patel T. A. Zech J. M. Cougle J. R. (2025). Two studies on the role of appearance concerns in social anxiety and depression. Behav. Ther. 56, 1082–1095. doi: 10.1016/j.beth.2025.05.002, 41139104

[ref40] Ramos-Jiménez A. Hernández-Torres R. P. Urquidez-Romero R. Wall-Medrano A. Villalobos-Molina R. (2017). Body image satisfaction as a physical activity indicator in university students. Am. J. Health Behav. 41, 599–607. doi: 10.5993/AJHB.41.5.9, 28760182

[ref41] Resnick B. Jenkins L. S. (2000). Testing the reliability and validity of the self-efficacy for exercise scale. Nurs. Res. 49, 154–159. doi: 10.1097/00006199-200005000-00007, 10882320

[ref42] Rhodes R. E. Martin A. D. Taunton J. E. Rhodes E. C. Donnelly M. Elliot J. (1999). Factors associated with exercise adherence among older adults. Sports Med. 28, 397–411. doi: 10.2165/00007256-199928060-00003, 10623983

[ref43] Ruan J. Yu R. Zhao Y. Xie L. Mei Y. (2025). Body talk on social networking sites and appearance anxiety among college students: the mediating role of self-objectification and moderating role of gender. Front. Psychol. 16, 1513923. doi: 10.3389/fpsyg.2025.1513923, 40276661 PMC12020388

[ref44] Sabiston C. M. Pila E. Vani M. Thogersen-Ntoumani C. (2019). Body image, physical activity, and sport: a scoping review. Psychol. Sport Exerc. 42, 48–57. doi: 10.1016/j.psychsport.2018.12.010

[ref45] Sheng J. Ariffin I. A. B. Tham J. (2025). The influence of exercise self-efficacy and gender on the relationship between exercise motivation and physical activity in college students. Sci. Rep. 15:11888. doi: 10.1038/s41598-025-95704-5, 40195417 PMC11977198

[ref46] Song H. Zeng W. Zeng T. (2022). Modeling community residents’ exercise adherence and life satisfaction: an application of the influence of the reference group. Int. J. Environ. Res. Public Health 19, 13174. doi: 10.3390/ijerph192013174, 36293754 PMC9603160

[ref47] Song S. H. Zhu Y. Q. (2019). The relationship between social media use and women’s negative body image [in Chinese]. Contemp. Commun., 2019, 29–34.

[ref48] Soulliard Z. A. Kauffman A. A. Fitterman-Harris H. F. Perry J. E. Ross M. J. (2019). Examining positive body image, sport confidence, flow state, and subjective performance among student athletes and non-athletes. Body Image 28, 93–100. doi: 10.1016/j.bodyim.2018.12.009, 30623802

[ref49] Sun Y. Zhao Y. Yang J. (2025). The impact of sports preferences on physical activity participation among college students: the mediating role of sports achievement emotions and exercise motivation. Front. Psychol. 16, 1565998. doi: 10.3389/fpsyg.2025.1565998, 40357484 PMC12066791

[ref50] Taylor M. K. Gill D. L. (2004). The psychology of exercise: integrating theory and practice. Sport Psychol. 18, 231–232. doi: 10.1123/tsp.18.2.231, 42027936

[ref51] Turel T. Jameson M. Gitimu P. Rowlands Z. Mincher J. Pohle-Krauza R. (2018). Disordered eating: influence of body image, sociocultural attitudes, appearance anxiety and depression - a focus on college males and a gender comparison. Cogent Psychol. 5:1483062. doi: 10.1080/23311908.2018.1483062

[ref52] Xue K. Yu M. Y. (2022). A Sociological Reflection on Appearance Anxiety [in Chinese]. People’s Tribune, 120–122.

[ref53] Yan Y. Zhou X. Zhou J. Chen Y. Zhang Y. Zhou X. . (2025). The relationship between facial negative physical self and social anxiety in college students: the role of rumination and self-compassion. Front. Psychol. 16, 1450174. doi: 10.3389/fpsyg.2025.1450174, 40567874 PMC12188541

[ref54] Zakaria R. Amor H. Baali A. (2022). Body image perceptions and avoidance behaviours among a moroccan group of adolescents. Ann. Hum. Biol. 49, 116–123. doi: 10.1080/03014460.2022.2072524, 35499239

[ref55] Zhang Y. Li L. (2021). The impact of sports participation on subjective well-being: evidence from sociological empirical research [in Chinese]. J. Shenyang Sport Univ. 40, 92–102, 117.

[ref56] Zhao Y. S. Ma Q. S. Li X. Y. Guo K. L. Chao L. (2023). The relationship between exercise motivation and exercise behavior in college students: the chain-mediated role of exercise climate and exercise self-efficacy. Front. Psychol. 14:1130654. doi: 10.3389/fpsyg.2023.1130654, 37063524 PMC10102370

[ref57] Zhou F. Wang W. Wu J. Nie Y. Shao C. Qiu W. . (2025). Body image and loneliness as mediators of the relationship between physical activity and exercise self-efficacy in college students. Sci. Rep. 15:30782. doi: 10.1038/s41598-025-16307-8, 40841721 PMC12371016

